# Latent profile analysis and influence factors study of presenteeism among ICU nurses in China

**DOI:** 10.3389/fpsyg.2023.1259333

**Published:** 2023-10-31

**Authors:** Yuxin Li, Jijun Wu, Xiaoli Liu, Jiquan Zhang, Xiaoli Zhong, Lin He

**Affiliations:** ^1^School of Nursing, North Sichuan Medical College, Nanchong, China; ^2^Department of Cardiology, Deyang People’s Hospital, Deyang, China; ^3^Department of Critical Care Medicine, Deyang People’s Hospital, Deyang, China; ^4^Department of Nursing, Deyang People’s Hospital, Deyang, China

**Keywords:** ICU nurses, presenteeism, perceived social support, latent profile analysis, influencing factors, China

## Abstract

**Background:**

Presenteeism is a significant global public health problem, and nurses are a high-prevalence group of presenteeism, affecting not only nurses’ physical and mental health, work efficiency, and quality of work but even poses a serious threat to patient safety.

**Objective:**

The categorization of presenteeism among ICU nurses is unclear. Our research aims to explore the subtypes of presenteeism among ICU nurses based on latent profile analysis, analyze the influencing factors of different subtypes, and provide a reference basis for developing targeted interventions to reduce the presenteeism rate.

**Methods:**

From January to February 2023, 509 ICU nurses in Sichuan Province, China, were selected as respondents and surveyed using the general information questionnaire, the presenteeism scale, and the perceived social support scale. Potential categories of presenteeism among ICU nurses were explored using potential profile analysis, and factors influencing the potential types of presenteeism among ICU nurses were investigated using the chi-square test and multivariate logistic regression analysis.

**Results:**

The best model was suggested to consist of three profiles: low presenteeism-normal coping group (18.3%), moderate presenteeism group (47.9%), and high presenteeism-work limitation group (33.8%). Multiple logistic regression results showed that secondary hospitals (*OR* = 0.116, *p* = 0.015), good physical health (*OR* = 0.084, *p* = 0.023), general physical health (*OR* = 0.016, *p* = 0.037), ICU human resource allocation = 1:2.5 to 3 (*OR* = 0.315, *p* = 0.007), and higher social support scores (*OR* = 0.975, *p* = 0.047) were more likely to be grouped into low presenteeism-normal coping group; married with no children (*OR* = 24.554, *p* = 0.005) were more likely to be grouped into moderate presenteeism group; and having experienced workplace violence in the past year (*OR* = 1.182, *p* = 0.049) were more likely to be grouped into high presenteeism-work limitation group.

**Conclusion:**

There is group heterogeneity in the presenteeism of ICU nurses, and nursing managers should develop targeted interventions to reduce the presenteeism rate of ICU nurses according to the characteristics and influencing factors of each type of presenteeism of ICU nurses.

## 1. Introduction

With the development of medical and health care and the continuous innovation of clinical nursing models, modern medical care has put forward higher requirements for nursing work. However, the shortage of nurses’ human resources, increased workload, and nurse–patient conflicts seriously affect nurses’ physical and mental health (Turpin et al., 2004; Mosadeghrad, 2013; Dhaini et al., 2017). Presenteeism, also known as impaired health productivity, was first proposed by Prof. Copper in 1996 and refers to the behavior of an employee who, although on duty, is unable to effectively engage in work due to physiological or psychological factors, which manifests itself in low productivity and reduces work engagement (Chapman, 2005; Rainbow et al., 2020). Presenteeism has become a common phenomenon in the workplace: studies have shown that 88% of organization employees have attended work when sick (McKevitt et al., 1997). Besides, in 2005, Government of Canada workers reported that more than 65.6 percent of employees worked when they were sick, and the average number of days they felt ill but had to work was 11.9 days per year (Biron et al., 2006). Due to the unique nature of nursing work with high pressure, high requirements, and high intensity, coupled with factors such as night shifts and poor work replaceability, nurses are more prone to presenteeism, and the incidence of presenteeism is four times higher than that of ordinary corporate employees (Lui and Johnston, 2019). Previous studies have shown that the presenteeism rate of nurses in Brazil is 32.8% (Silva-Costa et al., 2020), 39.52% in Korea (Baek et al., 2022), 49% in Sweden (Aronsson et al., 2000), 55% in Portuguese (Mosteiro-Díaz et al., 2020), and 94.25% in China (Shan et al., 2020). In terms of individual health and organizational productivity, presenteeism has more severe consequences than absenteeism due to illness. Nurses’ presenteeism behavior not only reduces productivity and quality of care but also increases the risk of medication errors and falls for patients and can even result in significant economic costs to society (Warren et al., 2011; Al Nuhait et al., 2017; Freeling et al., 2020). Studies show that the annual economic cost of nurse presenteeism behavior in North Carolina is between $2 and $13 billion (Letvak et al., 2012). Therefore, presenteeism behavior has become a significant global public health issue and, in recent years, has attracted the attention of multidisciplinary researchers in industrial and organizational psychology, occupational health psychology, and nursing management (Pilette, 2005; Allemann et al., 2019).

By the end of 2021, China’s nurse workforce reportedly reached 5.018 million, with 3.56 registered nurses per 1,000, but it has yet to reach the 2018 global level of 3.69 nurses per 1,000 population (World Health Organization, 2023). This shows that there is still a shortage of human resources for nurses in China. The intensive care unit (ICU) is a medical institution for emergency critical care patients’ rescue treatment of essential areas. Because of its closed management, ICU nurses need to continue to monitor the emergence of acute and critical patients with changes in the condition of the timely response to deal with the continued complexity of the essential patients, longer working hours, high standards of the work requirements, high-intensity workload, as well as closed and noisy work environment, so that the ICU nurses undergo a higher pressure and psychological burden than the other departments of the nurses, the phenomenon of presenteeism is also particularly prominent (Liang et al., 2017). Therefore, it is necessary to pay attention to the presenteeism of ICU nurses, analyze their influencing factors, and formulate targeted interventions, which are of great significance to promote the physical and mental health of ICU nurses and improve the quality of nursing care.

Studies have shown that social support is negatively correlated with presenteeism (Silva-Costa et al., 2020). That is, the higher the level of social support, the lower the presenteeism. Social support refers to the degree to which an individual is emotionally or materially connected to all aspects of society, from family, friends, and others (Langford et al., 1997). As an essential external resource, social support is vital to individuals’ physical and mental health. Perceived social support is a crucial component of social support. It refers to the extent to which individuals perceive support from various sources of social support, including individual family support, friend support, and other support (Blumenthal et al., 1987; Eisenberger et al., 2002). Theoretically, perceived social support may predict the occurrence of presenteeism through its effect on individual health. A study of presenteeism in a group of Chinese physicians found that the prevalence of presenteeism decreased by 8.3% for each unit increase in perceived social support availability (Xi et al., 2020). Research also suggests that the more social support nurses perceive from various sources of social support, the more beneficial it is to reduce the occurrence of presenteeism (Yang et al., 2019). The main effect and buffering effect model of social support state that social support has a general gainful effect on the development of individual mental health, buffering the adverse effects of stressful events on individuals’ physical and mental conditions and helping individuals to maintain good moods (Cohen and Wills, 1985; Uchino, 2006). The Job Requirements-Resources Model also points out that job requirements and resources are the core elements influencing an individual’s work engagement (Demerouti et al., 2001). As an essential work resource, social support can increase work engagement by stimulating internal and external motivation, incentivizing individuals to produce positive work outcomes, and buffering the negative consequences of illness, absenteeism, and presenteeism.

Latent profile analysis is a statistical method to determine the latent characteristics of survey respondents and typify them according to their response patterns on extraneous variables, which can obtain the category distribution proportions to study the population characteristics of each category further (Berlin et al., 2014; Sinha et al., 2021). In recent years, latent profile analysis has been widely used in nursing, management, and psychology. Still, there is no latent profile analysis of the presenteeism of ICU nurses. There are fewer studies on the relationship between presenteeism and social support of ICU nurses.

Therefore, this study used latent profile analysis to explore the potential profile characteristics of presenteeism of ICU nurses and its influencing factors, aiming to provide a reference basis for nursing managers to develop targeted interventions to reduce the presenteeism of ICU nurses.

## 2. Methods

### 2.1. Participants

This study was conducted in Sichuan Province, China, from January to February 2023, and 509 ICU nurses were finally included. There were 18 variables in this study, and the sample size was 108–216 according to the Kendall sample size rough estimation method, which selected at least 5–10 times the number of variables and considered 20% of invalid questionnaires (Ni et al., 2010). The inclusion criteria were (1) possession of a nurse practitioner’s license; (2) ≥1 year in ICU nursing; (3) informed consent and voluntary participation in this survey. The exclusion criteria were (1) internship, training, advanced training nurses; (2) absent nurses on maternity leave, personal leave, sick leave, etc. A total of 580 questionnaires were returned in this study, and 71 questionnaires with evident regular filling or logical errors and filling time ≤ 180 s were excluded, 509 valid questionnaires were recovered, and the valid questionnaire recovery rate was 87.8%.

### 2.2. Procedures

All the data in this study were collected in electronic questionnaires prepared by a questionnaire platform (Wen Juan Xing, wjx.cn). With the support of the director of the nursing department of each hospital, each hospital identified an ICU nurse manager as the survey liaison for this study to clarify the purpose and significance of this survey. To ensure the reliability of the questionnaire data, all the items were set as compulsory answers and could be submitted only after they were all completed.

### 2.3. Measures

#### 2.3.1. Socio-demographic information

The socio-demographic information consisted of two parts: the personal-contextual of the ICU nurses, including gender, age, marital and childbearing status, highest education, physical health status, presence of chronic diseases, and presence of night shifts; and the work-contextual, including the hospital level, professional title, position, years of ICU work, income per month, ICU human resource allocation, and exposure to workplace violence in the past year.

#### 2.3.2. Stanford presenteeism scale

The Stanford Presenteeism Scale developed by Koopman et al. (2002) and Chineseized by Zhao et al. (2010) was used to assess nurses’ impaired productivity due to health problems. The scale consists of six items, including two dimensions of completing work and avoiding distractions; items 5 and 6 are reverse-scored. The scale was based on a 5-point scale, with scores ranging from 1 to 5, from complete disagreement to complete agreement, where item 5 and item 6 were reverse scored, and a total score of 6 to 30, with higher scores indicating more severe presenteeism. The Cronbach’s alpha coefficient for this scale was 0.81.

#### 2.3.3. Perceived social support scale

Individuals’ perceived level of support from various sources of social support was assessed using the perceived social support scale developed by Blumenthal et al. (1987) and Chineseized by Jiang (2001). The scale consists of 3 dimensions and 12 entries, including family support, friend support, and other support. A 7-point scale was used, with scores ranging from 1 to 7, from strongly disagree to agree strongly, and a total score of 12–84, with higher scores indicating higher levels of social support. The Cronbach alpha coefficient of the scale was 0.954.

### 2.4. Statistical analysis

Mplus 8.3 software was used to establish the latent profile classification model. Considering that the item scores reflect the dimension scores and that the item scores are more reflective of the characteristics of the individual item scores, the ICU nurses’ latent presenteeism scale item scores were used as the model exogenous variables, and the number of categorical models was gradually increased starting from the initial model. The model fit was judged based on the Akaike information criterion (AIC), Bayesian information criterion (BIC), and sample-size-adjusted BIC (aBIC), with smaller values representing a better fit; Bootstrapped likelihood ratio test (BLRT) and Lo–Mendell–Rubin (LMR) were used to compare the fit differences among the models. The information entropy is between 0 and 1, and the entropy value >0.8 and closer to 1 indicates that the model is more accurate in categorization; LMR and BLRT are used to compare the fitting differences between the k-category and k-1-category models, and when the difference is statistically significant (*p* < 0.05), it indicates that the k-category profile model is better than the k-1-category profile model. After determining the best potential profile model, SPSS 26.0 software was used to analyze the data, and the mean ± standard deviation was used to describe the measurement data that conformed to the normal distribution; the count data were expressed as frequencies and percentages. Comparisons between groups were made employing the chi-square test, Fisher’s exact probability method, and analysis of variance. The factors with significant differences in the results of the univariate analysis were used as independent variables, the continuous numerical variables and total scale scores were used as covariates, and the results of potential profile analysis of presenteeism of ICU nurses were used as dependent variables to conduct multiple logistic regression analysis to explore the factors influencing the classification of the possible profile of presenteeism of ICU nurses. The difference was considered statistically significant at *p* < 0.05.

### 2.5. Ethical considerations

Our research was in accordance with the ethical standards formulated in the Declaration of Helsinki and was confirmed by the Deyang People’s Hospital ethics committee approval (2021–04-056-K01). Informed consent was provided by the participants prior to their participation. The survey was anonymous, and the confidentiality of the information was assured.

## 3. Results

### 3.1. Socio-demographic characteristics of the participants

Among the 509 ICU nurses, 40 cases (7.9%) were male and 468 (92.1%) were female; age: 191 cases (37.5%) ≤ 29 years old, 281 cases (55.2%) 30–39 years old, 31 cases (6.1%) 40–49 years old, 6 cases (1.2%) ≥ 50 years old; marital status: 152 cases (29.9%) unmarried, 48 cases (9.4%) married with no children, 300 cases (9.8%) married with children, 9 cases divorced/widowed. Table 1 displays all of the remaining general data.

**Table 1:** Comparison of socio-demographic characteristics among different presenteeism of ICU nurses profiles (*n* = 509).

Variables		Low presenteeism-normal coping group (*n* = 93)	Moderate presenteeism group (*n* = 244)	High presenteeism-work limitation group (*n* = 172)	*χ* ^2/F^	*p*
Gender	Male	8(8.6%)	18 (7.4%)	14 (8.1%)	0.168	0.919
	Female	85 (91.4%)	226 (92.6%)	158 (91.9%)		
Age	≤29	37 (39.8%)	89 (36.5%)	65 (37.8%)	3.638	0.729
	30 ~ 39	50 (53.8%)	132 (54.1%)	99 (57.6%)		
	40 ~ 49	5 (5.4%)	19 (7.8%)	7 (4.1%)		
	≥50	1 (1.1%)	4 (1.6%)	1 (0.6%)		
Marital or childbearing status	Unmarried	34 (36.6%)	62 (25.4%)	56 (32.6%)	16.970	0.007
	Married with no children	2 (2.2%)	26 (10.7%)	20 (11.6%)		
	Married with children	53 (57%)	154 (63.1%)	93 (54.1%)		
	Divorced/widowed	4 (4.3%)	2 (0.8%)	3 (1.7%)		
Hospital level	Secondary hospital	7 (7.5%)	16 (6.6%)	2 (1.2%)	7.953	0.019
	Tertiary hospital	86 (92.5%)	228 (93.4%)	170 (98.8%)		
Highest education	Junior college and below	19 (20.4%)	37 (15.2%)	23 (13.4%)	3.921	0.398
	Undergraduate	72 (77.4%)	205 (84.0%)	146 (84.9%)		
	Master degree or above	2 (2.2%)	2 (0.8%)	3 (1.7%)		
Professional title	Nurse	16 (17.2%)	36 (14.8%)	25 (14.5%)	7.677	0.263
	Nurse Practitioner	49 (52.7%)	114 (46.7%)	96 (55.8%)		
	Nurse Supervisor	22 (23.7%)	83 (34.0%)	47 (27.3%)		
	Deputy chief nurse and above	6 (6.5%)	11 (4.5%)	4 (2.3%)		
Position	Clinical Nurse	72 (77.4%)	192 (78.7%)	147 (85.5%)	4.865	0.301
	Nursing team leader	13 (14.0%)	34 (13.9%)	19 (11.0%)		
	Head Nurse	8 (8.6%)	18 (7.4%)	6 (3.5%)		
Years of ICU work	<6	37 (39.8%)	96 (39.3%)	57 (33.1%)	4.121	0.390
	6 ~ 10	34 (36.6%)	77 (31.6%)	69 (40.1%)		
	>10	22 (23.7%)	71 (29.1%)	46 (26.7%)		
Income per month	<6,000	23 (24.7%)	51 (20.9%)	27 (15.7%)	7.078	0.314
	6,001 ~ 8,000	44 (47.3%)	105 (43.0%)	90 (52.3%)		
	8,001 ~ 11,000	20 (21.5%)	62 (25.4%)	36 (20.9%)		
	>11,001	6 (6.5%)	26 (10.7%)	19 (11.0%)		
Physical health condition	Good	59 (63.4%)	150 (61.5%)	63 (36.6%)	58.100	<0.001
	General	33 (35.5%)	91 (37.3%)	81 (47.1%)		
	Worse	1 (1.1%)	3 (1.2%)	28 (16.3%)		
Suffering from chronic disease	Yes	11 (11.8%)	37 (15.2%)	45 (26.2%)	11.336	0.003
	No	82 (88.2%)	207 (84.8%)	127 (73.8%)		
ICU human resource allocation	<1:2.5 ~ 3	44 (47.3%)	153 (62.7%)	99 (57.6%)	20.088	<0.001
	=1:2.5 ~ 3	36 (38.7%)	59 (24.2%)	32 (18.6%)		
	>1:2.5 ~ 3	13 (14.0%)	32 (13.1%)	41 (23.8%)		
Night shift	Yes	76 (81.7%)	207 (84.8%)	155 (90.1%)	4.121	0.127
	No	17 (18.3%)	37 (15.2%)	17 (9.9%)		
Experienced workplace violence in the past year	Yes	26 (28.0%)	71 (29.1%)	84 (48.8%)	20.024	<0.001
	No	67 (72.0%)	173 (70.9%)	88 (51.2%)		
Perceived social support score		63.55 ± 11.09	65.59 ± 10.84	58.24 ± 12.14	21.474	<0.001

### 3.2. Latent profiles analysis of presenteeism

Individual-centered potential profile analysis was performed on the presenteeism scores of 509 ICU nurses, and latent profile models for categories 1–4 were fitted sequentially, as shown in Table 2. As the number of categories increased, the AIC, BIC, and aBIC gradually decreased, and when the number of categories was 3, the entropy value was 0.832, and the LMR and BLRT values were statistically significant (*p* < 0.05); when the number of categories was 4, the LMR value is not statistically significant (*p* > 0.05), so model 3 is determined as the optimal latent profile analysis model by careful consideration.

**Table 2:** Model fit indices of latent profile analysis of presenteeism (*n* = 509).

Model	AIC	BIC	aBIC	Entropy	*p*-value		Latent class probability
					LMR	BLRT	
1 class	9100.690	9151.479	9113.389	–	–	–	–
2 class	8353.436	8433.853	8373.544	0.872	<0.001	<0.001	0.660/0.340
3 class	8194.450	8304.494	8221.967	0.832	0.001	<0.001	0.183/0.479/0.338
4 class	8036.642	8176.312	8071.566	0.795	0.105	<0.001	0.358/0.147/0.289/0.206

**Figure 1:**
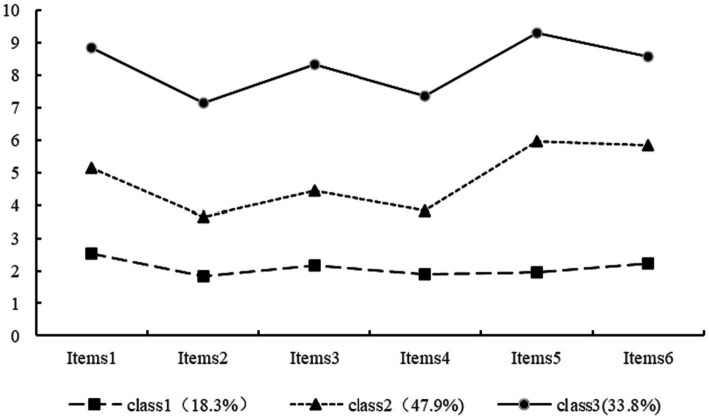
The distribution of three potential classes of presenteeism.

Model 3 was used as the ideal model to name the categories based on the scores of the 3 categories of ICU nurses on the 6 items, and a chart was drawn based on the scores of the 3 categories of presenteeism of ICU nurses on the 6 items (see Figure 1). Class 1 accounted for 18.3% of the total, and the scores on the items for class 1 were low. The low scores for items 2 and 4, with item 2 (“I cannot complete difficult tasks at work because of health problems”) belonging to the completing work dimension and item 4 (“I do not think it’s possible to carry out certain tasks at work because of health problems)” belongs to the avoiding distractions dimension, reflecting that this category of ICU nurses has a low level of presenteeism and can cope with work tasks normally, so it was named the “low presenteeism-normal coping group.” Class 2 accounted for 47.9% of the total number of people, and the scores of each item of presenteeism were at a medium level, so it was named the moderate presenteeism group. Class 3, with a share of 33.8%, had the highest score for each item in this category. Among them, item 1 (“It is more difficult to regulate my stress at work due to health problems”) belongs to the avoiding distractions dimension, which scored high; items 5 and 6 scored high (“Despite health problems, I am still able to concentrate on getting my work done “; “Despite health problems, I still feel energized to do all my work”) belong to the completing work dimension, reflecting the high level of presenteeism and limited work energy of this group of ICU nurses. Therefore, it was named the “high presenteeism-work limitation group.”

### 3.3. Predictor of latent profile membership

The chi-square test and one-way analysis of variance showed that the ICU nurses in the three groups had statistically significant (*p* < 0.05) scores for marital status, hospital level, physical health status, presence of chronic diseases, ICU human resource allocation, whether they had experienced workplace violence in the past year, and perceived social support, as shown in Table 1.

Using the latent category of presenteeism of ICU nurses as the dependent variable (the “low presenteeism-normal coping group “as the reference) and the statistically significant factors in the univariate analysis as the independent variables, the results of multiple logistic regression analysis showed that marital status, hospital level, physical health status, human resource allocation, whether they had experienced workplace violence in the past year, and perceived social support were influential factors affecting the potential profile of implicit absence of ICU nurses (*p* < 0.05). The presence of a chronic disease was not an influential factor influencing the potential profile of presenteeism among ICU nurses (*p* > 0.05). The results of multiple logistic regression are shown in Table 3. in which secondary hospitals (*OR* = 0.116, *p* = 0.015), good physical health (*OR* = 0.084, *p* = 0.023), general physical health (*OR* = 0.016, *p* = 0.037), ICU human resource allocation = 1:2.5 to 3 (*OR* = 0.315, *p* = 0.007), and higher perceived social support scores (*OR* = 0.975, *p* = 0.047) were more likely to be grouped into low presenteeism-normal coping group; married with no children (*OR* = 24.554, *p* = 0.005) were more likely to be grouped into moderate presenteeism group; and having experienced workplace violence in the past year (*OR* = 1.182, *p* = 0.049) were more likely to be grouped into high presenteeism-work limitation group.

**Table 3:** The multifactor analysis of presenteeism of ICU nurses by logistic regression.

Variable		Moderate presenteeism group				High presenteeism-work limitation group			
		*β*	OR	95%CI	*P*	*β*	OR	95%CI	*P*
Marital or childbearing status (take divorced/widowed as reference)	Unmarried	1.304	3.685	(0.617,22.001)	0.153	0.432	1.540	(0.252,9.405)	0.640
	Married with no children	3.201	24.554	(2.545,236.904)	0.006	2.240	9.394	(0.252,9.405)	0.057
	Married with children	1.709	5.525	(0.948,32.208)	0.057	0.592	1.807	(0.304,10.740)	0.515
Hospital level (take tertiary hospital as reference)	Secondary hospital	−0.371	0.690	(0.259,1.840)	0.458	−2.144	0.117	(0.021,0.663)	0.015
Physical health condition (take worse as reference)	Good	−0.034	0.967	(0.093,10.042)	0.978	−2.791	0.061	(0.007,0.511)	0.010
	General	0.006	1.006	(0.097,10.481)	0.996	−2.246	0.106	(0.013,0.877)	0.037
Suffering from chronic disease (take no as reference)	Yes	0.417	1.518	(0.698,3.298)	0.292	0.273	1.314	(0.576,2.998)	0.516
Experienced workplace violence in the past year (take no as reference)	Yes	0.081	1.084	(0.621,1.892)	0.776	0.595	1.812	(1.002,3.279)	0.049
ICU human resource allocation (take >1:2.5 ~ 3 as reference)	<1:2.5 ~ 3	0.284	1.328	(0.632,2.794)	0.454	−0.239	0.787	(0.366,1.693)	0.540
	=1:2.5 ~ 3	−0.411	0.663	(0.303,1.451)	0.304	−1.190	0.304	(0.132,0.701)	0.005
Perceived social support score		0.020	1.020	(0.997,1.044)	0.090	−0.025	0.975	(0.952,1.000)	0.047

Reference group: low presenteeism-normal coping group. OR, Odds ratio; 95% CI, 95% Confidence Interval.

## 4. Discussion

In this study, using latent profile analysis, it was found that there was significant individual variability in the presenteeism of ICU nurses, which could be divided into 3 latent profiles: low presenteeism-normal coping group, moderate presenteeism group, and high presenteeism-work limitation group, accounting for 18.3, 47.9, and 33.8%, respectively. Among them, ICU nurses in the low presenteeism-normal coping group were mainly characterized by good physical condition, not suffering from chronic diseases, and not having suffered workplace violence in the past year. ICU nurses in the high presenteeism-work limitation group were mainly characterized by being married with children, working in a tertiary hospital, and having an ICU human resource allocation of <1:2.5 ~ 3. The sum of the 2 profiles of moderate and high presenteeism groups accounted for 81.7%, indicating that the presenteeism of ICU nurses was at a medium to a high level, which was higher than the results of Zhang et al. (2023). The analysis of the reason may be related to the different study subjects. The former study subjects came from the clinical nurses of comprehensive departments. In contrast, the subject of this study was ICU nurses, who were under high pressure and in an overloaded working environment for a long time, so the presenteeism was more serious.

This study found that ICU nurses in secondary hospitals were more likely to be in the “low presenteeism-normal coping group.” Related studies also confirmed that hospital level was closely related to nurses’ presenteeism, and the higher the hospital level was, the more likely nurses were to have presenteeism (Ren et al., 2019). The reason maybe that compared with tertiary hospitals, the number of patients in secondary hospitals is smaller, the disease is relatively milder, and the work pressure and workload of nurses are less, while ICU nurses in tertiary hospitals not only have to undertake medical treatment tasks but also have to undertake more research and clinical teaching tasks, so under the tight human resource situation in tertiary hospitals, the overload of work is more likely to aggravate the presenteeism of ICU nurses.

Good and general health were more likely to be in the “low presenteeism-normal coping group, “which is consistent with the findings of Fiorini et al. (2022). The study also pointed out that the physical condition of nurses had a significant adverse effect on presenteeism (Martinez and Ferreira, 2012; Whysall et al., 2018; Shan et al., 2022). That is, the worse the physical health condition is, the more serious the presenteeism is, and ICU nurses were more likely to suffer from chronic diseases such as cervical spondylosis and lumbar disc herniation during long and intensive working hours, which increased the risk of presenteeism (Dhaini et al., 2017; Santos et al., 2018). Nursing managers should pay attention to ICU nurses’ physical and mental health status, appropriately reduce the workload of nurses with poor physical health status, and give adequate humanistic care.

ICU human resource allocation = 1:2.5 to 3 is more likely to belong to the “low presenteeism-normal coping group.” A reasonable human resource allocation in the ICU can effectively reduce the presenteeism rate of nurses. The study pointed out that due to the lack of sufficient human resources, nurses chose to work with illness to avoid increasing the workload of their colleagues, which increased the presenteeism rate (Dhaini et al., 2016). Nursing managers should reasonably allocate human resources according to the actual situation of the department, adjust the bed-nurse ratio, reduce nurses’ workload, reduce the presenteeism rate, and improve the quality of nursing services.

Married ICU nurses with no children were 24.554 times more likely to be in the “moderate presenteeism group” than the “low presenteeism-normal coping group.” Several studies have pointed out that marital status is the main influencing factor on nurses’ presenteeism, especially for married nurse’s multiple roles in the family (Shan et al., 2020; Gillet et al., 2021; Shan et al., 2022). When they need to cope with work situations simultaneously, it is often challenging to balance work and family, which affects clinical work efficiency and increases presenteeism. It is suggested that nursing managers should pay attention to the psychological and employment status of married nurses, and reasonably flexible scheduling should be combined with the individualized situation of married nurses to help nurses better balance family and work and reduce the presenteeism rate.

Nurses who have suffered from workplace violence in the past year are more likely to belong to the group of high presenteeism-work limitation group. The reason is that nurses are prone to verbal or behavioral violence from patients and family members in the work process. Workplace violence can cause nurses to suffer from physical and mental pain, resulting in negative emotions and burnout, lowering their sense of value and professional identity and thus triggering presenteeism (Yang et al., 2015). Therefore, nursing managers should provide timely psychological counseling to nurses who have been subjected to workplace violence and reasonably transfer them to other jobs; in addition, they should actively improve the hospital security system to enhance nurses’ sense of job security and thus reduce presenteeism.

Higher perceived social support scores were more likely to be categorized in the low presenteeism-normal coping group, consistent with previous studies (Liu et al., 2022). Perceived social support is the degree to which an individual perceives that support is available from family, friends, and other outside sources. In general, employees lacking social support are more likely to experience presenteeism, and supportive colleagues and leaders may encourage employees to stay home when they are sick (Miraglia and Johns, 2016; Whysall et al., 2018). As a work resource, social support from leaders and coworkers is conducive to improving work efficiency, enhancing job satisfaction and a sense of professional identity, and reducing presenteeism. In addition, care from family and friends can reduce the physical and mental burden, buffer the negative impact of stressful events on individuals’ physical and mental conditions, help individuals maintain good moods, promote healthy productivity, and reduce presenteeism (Coffeng et al., 2014; Kim and Ryu, 2015). Therefore, nursing managers should focus on ICU nurses with low levels of social support and create a supportive work environment for them to feel the care and support of the organization. In addition, encouraging the creation of a harmonious family atmosphere and building a supportive family environment provides nurses with spiritual support and assistance to reduce the incidence of presenteeism.

## 5. Limitations

This study has several limitations. First, this study is cross-sectional, which limits its ability to make causal arguments. Second, this study only selected ICU nurses in Sichuan Province, China, as the study population, which has some limitations in the sample; the next step requires a multicenter, large-sample survey study to explore the characteristics and influencing factors of presenteeism among ICU nurses in different regional hospitals.

## 6. Conclusion

In this study, the presenteeism characteristics of ICU nurses were classified into three categories: “low presenteeism-normal coping group,” “moderate presenteeism group,” and “high presenteeism-work limitation group” by potential profile analysis. There are differences in the distribution of marital status, hospital level, physical health status, ICU human resources allocation, and social support among different presenteeism categories. ICU nurses were more likely to be in good physical health, to be located in a secondary hospital, to have a reasonable allocation of ICU human resources, and to have a high level of social support categorized as being in the low presenteeism group; more likely to be married with no children classified as being in the moderate presenteeism group; and more likely to have experienced workplace violence in the past year categorized as being in the high presenteeism group. Therefore, it is suggested that nursing managers focus on nurses with moderate and high presenteeism and strengthen the prevention and intervention of presenteeism for nurses. For example, managers should give full humanistic care to ICU nurses who are married and have children, rationally allocate human resources according to the actual situation of the department, and implement flexible scheduling. In addition, they should create a favorable working environment for ICU nurses and enhance social support to reduce the presenteeism rate of ICU nurses.

## Data availability statement

The original contributions presented in the study are included in the article/Supplementary material, further inquiries can be directed to the corresponding authors.

## Ethics statement

The studies involving humans were approved by the Deyang People’s Hospital, Sichuan, China. The studies were conducted in accordance with the local legislation and institutional requirements. The participants provided their written informed consent to participate in this study.

## Author contributions

YL: Data curation, Formal analysis, Investigation, Software, Validation, Writing – original draft, Writing – review & editing. JW: Data curation, Formal analysis, Investigation, Methodology, Software, Validation, Writing – original draft, Writing – review & editing. XL: Data curation, Formal analysis, Investigation, Supervision, Validation, Writing – review & editing. JZ: Data curation, Formal analysis, Investigation, Software, Writing – review & editing. XZ: Formal Analysis, Project administration, Resources, Supervision, Validation, Writing – review & editing. LH: Project administration, Resources, Supervision, Validation, Visualization, Writing – review & editing.
